# Comparative transcriptome analysis of wheat in response to corn leaf aphid, *Rhopalosiphum maidis* F. infestation

**DOI:** 10.3389/fpls.2022.989365

**Published:** 2022-11-24

**Authors:** Poonam Jasrotia, Samriti Sharma, Mohini Nagpal, Disha Kamboj, Prem Lal Kashyap, Satish Kumar, Chandra Nath Mishra, Sudheer Kumar, Gyanendra Pratap Singh

**Affiliations:** ^1^ Division of Crop Protection, ICAR- Indian Institute of Wheat and Barley Research, Karnal, Haryana, India; ^2^ Division of Crop Improvement, ICAR- Indian Institute of Wheat and Barley Research, Karnal, Haryana, India

**Keywords:** cereals, aphids, host plant resistance, transcriptome, plant defense

## Abstract

Aphids are one of the most important insect pests of wheat crop in all wheat growing regions of the world. Amongst various aphid species, the corn leaf aphid (*Rhopalosiphum maidis* F.) is considered one of the most destructive insect pests of wheat in the North Western Plains region of India. Transcriptome profiling of highly susceptible wheat *Triticum durum* genotype, A-9-30-1 and tolerant wheat *Triticum aestivum* genotype, HD2967 was performed to investigate aphid-host interactions. The results obtained from differential gene expression analysis of *R. maidis* on the highly susceptible genotype, A-9-30-1 plants, when compared with the tolerant genotype, HD2967, showed that 212 genes were significantly upregulated and 1009 genes were significantly downregulated. Our findings demonstrated that the genes associated with defense were significantly higher in response to *R. maidis* on HD2967 as compared to A-9-30-1. Additionally, various genes with physiological attributes were expressed during aphid attack. Based on gene ontology classification, three classifications, such as, cellular components (CC), molecular function (MF), and biological processes (BP) of sequences were identified. KEGG enrichment analysis revealed that twenty-five pathway genes were differentially expressed during the infestation of wheat with *R. maidis*. Notable changes were observed in A-9-30-1 and HD2967 transcriptomic profiling after infestation. The results obtained in the present study will help to elucidate the mechanism governing host-pest interaction and may lead to the development of new methods for increasing the resistance level of wheat against *R. maidis*, including over-expression of defense-related genes.

## Introduction

Plants interact with various insect pests and develop complex defense mechanisms to counter their attacks. They can perceive herbivory-associated molecular patterns (HAMPs) or damage-associated molecular patterns (DAMPs) incited by insects and mount direct or indirect defense responses against them (Howe and Jander, 2008; Bonaventure, 2012). Direct defense response includes the production and accumulation of defensive chemicals, i.e. proteinase inhibitors, plant secondary metabolites (PSMs), polyphenol oxidases, and other defensive proteins, which are produced by herbivory thereby lowering their performance (Steppuhn and Baldwin, 2007; Hartl et al., 2010; War et al., 2012; Singh et al., 2021). Indirect defense responses constitute traits that themselves do not have direct effect on herbivores. However, these responses lead to the biosynthesis of complex blends of volatiles that can attract natural enemies (parasitoids and predators) of the herbivores and thereby, reduce plant loss (Pare and Tumlinson, 1999; Kessler and Baldwin, 2002). Plants activate different defensive strategies when they are attacked by insect pests (Afroz et al., 2021; Mertens et al., 2021). Insect pests belonging to a particular insect order category produce a specific type of response in attacked plants. For instance, when leaf-eating beetles (Coleoptera) or caterpillars (Lepidoptera) attack plants, they cause extensive tissue damage, which mainly activates the Jasmonic acid (JA)-mediated defense pathway in plants (Moreira et al., 2018; Yang et al., 2019). On the contrary, the piercing-sucking hemipteran insects such as whiteflies and aphids feed on the phloem sap through modified mouthparts (stylets). In response to feeding by piercing and sucking hemipteran insects, plants activate salicylic acid (SA) pathways causing less damage to plant cells in comparison to leaf-eating insects (Jiang et al., 2019; Costarelli et al., 2020).

Corn leaf aphid, *Rhopalosiphum maidis* is basically a serious insect pest of maize crop but it also attacks wheat crop, and is distributed throughout the world i.e. from tropics to temperate regions causing around 25 to 30% yield loss under severe infestation (Chandrasekhar et al., 2018; Jasrotia et al., 2021). Aphids are polyphagous pests and cause damage not only to one crop but also attack weeds from the gramineae, cyperaceae, and typhaceae families. Aphids cause direct damage by sucking cell sap from leaves and young shoots, causing distortion, stunting, leaf curling, wilting, and twisting. Indirect damage by aphids leads to the deposition of honeydew that reduces photosynthetic activity and induces sooty mold production and premature leaf senescence (Singh and Jasrotia, 2020). Aphids present a challenge to agricultural crops because of their short life span and high dispersion rates. Therefore, enormous amounts of pesticides are being used, and these are costly and destroy non-targeted beneficial natural enemies (Jasrotia & Katare, 2018) and also lead to high levels of pest resistance and resurgence. As an alternative to chemical control, host plant resistance is considered an eco-friendly pest management approach and serves as an essential component of IPM programs. It is a heritable plant trait that has been described in several crops. Many aphid-resistant crop plants have been deployed for sustainable pest management during the past century (Smith and Chuang, 2014). Reduced insecticide applications, residues, and mortality of natural enemies and improved water quality are some of the ecological benefits arising from host plant resistance. However, knowledge of molecular mechanisms and genetics underlying aphid resistance is not yet elucidated or exploited. The impact of the resistance spectrum geographically on various aphid biotypes is also a point of consideration. In this direction, the rapid development of transcriptomic technologies will serve as a knowledge base for arthropod plant interactions and lead to immense use of aphid resistance in sustainable production systems. With this perspective, the present study was planned to analyze the transcriptional response of two contrasting genotypes, i.e., highly susceptible and tolerant hosts, at the time of aphid attack to study the genes and pathways modulated during aphid-host interaction.

## Materials and methods

### Experimental set-up for host plant resistance response studies

The studies were carried out under the glasshouse of ICAR-Indian Institute of Wheat and Barley Research (IIWBR), Karnal, Haryana, India during 2018. The seeds of chosen wheat *T. durum* genotype, A-9-30-1, and tolerant wheat *T. aestivum* genotype, HD2967 were obtained from the Germplasm Resources Unit (GRU) of ICAR-IIWBR, Karnal. For the study, each genotype was planted in a plastic pot containing a mixture of farmyard manure, vermicompost, and cocopeat (2:2:1). Corn leaf aphids (*R. maidis*) colonies were established by collecting aphids from wheat fields and rearing them on wheat susceptible genotype A-9-30-1 in a glass house. The aphids were maintained for several generations before the initiation of the studies to get a pure aphid population. Host resistance response was recorded by measuring nymphiposition, nymphal duration, and nymphal mortality of aphids on the susceptible genotype A-9-30-1 and the tolerant genotype HD2967. The host plant resistance response studies (nymphiposition, nymphal duration, and nymphal mortality) of *R. maidis* on both the genotypes HD2967 and A-9-30-1 were recorded during January-March, 2018.

To record nymphiposition, five adult aphids of *R. maidis* were released on each test genotype inside a glass chimney for 24 h to get pre-conditioned nymphs, and then the adult aphids were removed after the laying of young nymphs. These young ones were kept on each test genotype for 10 days until they underwent their last molt and attained a reasonable size. The aphids were transferred to a seven-day-old test plant of each genotype. The developing aphids were regularly observed and the numbers of nymphs deposited/plant/day were counted and removed daily. Nymph production was recorded until the death of the mother aphid. The total number of nymphs laid throughout the life of an aphid was counted, and plants were randomized by genotype with 10 replications. The number of nymphs deposited by each aphid till death was summed up. Mean fecundity and the number of nymphs/aphid/day were calculated.

For measuring the nymphal duration and their survival, five adult aphids were kept on seven-day-old plant of each tested genotype. After 12 h, the adult aphids were removed after nymphiposition on the tested plants, and only 10 neonate nymphs were allowed to grow, and the rest of the nymphs were removed. After 10 days of total feeding period, the number of nymphs survived and dead was recorded. The nymphal duration period was calculated from birth till the last molt or laying of first young one by an aphid adult.

### Sample collection for transcriptomic studies

At the two-leaf stage (12-day old plants), 20 apterous adult aphids of *R. maidis* were confined to the first leaf of wheat seeding of each test genotype (A-9-30-1 & HD2967) inside a glass chimney and were allowed to feed on test genotypes for 48 h. New-born nymphs produced by aphid adults were carefully removed every 12 h using a brush. After 48 h of feeding, all the aphids were removed, and leaf tissues of approximately 2.5 × 2.5 cm^2^ from the aphid feeding sites of each plant were harvested and flash frozen with liquid nitrogen for further processing of RNA extraction. Three leaf sections covering the aphid feeding sites were collected from three independent plants of each chosen genotype and pooled to form one biological replicate. Three biological replicates were performed for each treatment. Similarly, sample collection was also done from uninfested plants of each genotype that served as a control.

### Isolation of RNA, cDNA library construction, and sequencing

Total RNA was isolated from young infested and uninfested leaves of susceptible genotype, A-9-30-1 and tolerant genotype, HD2967 infested through the ZR (control) plant RNA miniprep (ZYMO Research) kit. The quality and quantities of the isolated RNA were checked on a 1% denaturing RNA agarose gel and Nanodrop (NanoDrop technology, USA), respectively. RNA samples having RIN values of more than 8.0 and an OD 260/280 ratio of more than 1.7 were used for RNA-sequencing. The isolated mRNAs were purified using oligo (dT) magnetic beads (Illumina, Inc) from total RNA.RNA-seq pair-end libraries were prepared using the Illumina TruSeq stranded mRNA sample prep kit. These short fragments of mRNA were used as templates for first-strand cDNA conversion using SuperScript II and Act-D mix to facilitate RNA-dependent synthesis for each sample (Hyun et al., 2014; Gutierrez et al., 2017). The first-strand cDNA was then used as a template strand for second-strand synthesis by using a second-strand mix. These cDNA fragments were then purified by using Ampure XP beads followed by A-tailing and adaptor ligation. The products of the ligation reaction were purified on agarose gel electrophoresis that was enriched by PCR for final cDNA library generation (Sharma et al., 2019). These PCR-enriched libraries were further analyzed in the 4200 tape station system of Agilent technologies by using D1000 screen tape (high sensitivity) for quality and quantity checks. The mean of the library distribution was 547 bp for A-9-30-1 (infested leaf), 555 bp for A-9-30-1 (un-infested leaf), 520 bp for HD2967 (infested leaf), and 512 bp for HD2967 (un-infested-leaf), respectively. These cDNA libraries were loaded onto the NextSeq 500 for cluster generation and sequencing. Pair-end sequencing allows the template fragments to be sequenced in both the forward and reverse directions on the NextSeq 500 sequencing platform (Illumina, Inc.) to obtain 2×100 bp paired-end reads.

### Preprocessing of raw Illumina data

For obtaining high-quality reads, reads with adapter sequences, ambiguous reads (reads with unknown nucleotides “N” larger than 5%), and low-quality reads (reads with more than 100% quality threshold (QV)<20 phred score) were removed by the Trimmomatic v0.35 software. FastQC was used for quality analysis of cleaned reads from all samples. After this, a reference genome index was set by using Bowtie2 software, whereas the remaining clean reads from paired-end sequencing were mapped to the reference genome (http://www.ensembl.org/index.html) through TopHat2. HtSeq software was used to count the read numbers mapped to every gene. Differential expression of genes was analyzed through the DESwq R package (http://www.bioconductor.org/packages/release/bioc/html/DESeq.html). The significant differences in gene expression were calculated by the absolute value of log_2_ fold change>1 and P-value<0.05. After an aphid attack, functional enrichment analyses such as Kyoto Encyclopedia of Genes and Genomes (KEGG) and Gene Ontology (GO) were used to identify the DEGs. The Blast2GO program was used for gene annotation against the Gene Ontology database (http://geneontology.org/) (Ivamoto et al., 2017). KEGG pathways against the KEGG database (http://www.genome.jp/kegg) with a P-value < 0.05 were analyzed, which were significantly deemed in the DEG analysis.

### Quantitative real-time PCR

For the validation of sequencing results, total RNA was isolated from young and healthy leaves of A-9-30-1 infested, A-9-30-1 uninfested, HD2967 infested, and HD2967 uninfested by using the Real genomics Hiyield™ total RNA mini kit (Real Biotech Corporation Ltd.). Then, the Thermo Scientific Revert Aid first-strand cDNA synthesis kit was used for the reverse transcription. Pathogenesis-related gene-specific primers were developed by using Primer3 software (Rozen and Skaletsky, 2000). The reactions of real-time PCR were carried out in triplicate using the ABI PRISM 7700 Real-time PCR system with the ABI 7700 Fast sequence detector (Applied Biosystems, USA). The expression of variant selected genes was normalized by the housekeeping gene ‘Actin’ of wheat. The designed primers (**Supplementary information: Table 1**) were tested for single-band amplification through conventional endpoint PCR. Melting curve analysis was performed using qPCR in a reaction containing 1X SYBR Green Master mix (Applied Biosystems, USA), 1 µl cDNA template, 10 pmol of each primer, and a final volume of 20 µl was prepared by adjusting with nuclease-free water (Sharma et al., 2019). The conditions that were followed for the real-time PCR experiment included initial denaturation at 95˚C for 30 s, followed by 35 cycles of denaturation at 95˚C for 10 s, and extension for 10 s based on primer Tm followed by a thermal dissociation curve. The relative expression level was analyzed using the 2^-ρρct^ method (Schmittgen and Livak, 2008).

### Data analysis

The data for aphid resistance identification among genotypes were analyzed by one-way analysis of variance (ANOVA, P < 0.05). The results of biological parameters i.e. nymphiposition, and nymphal duration and survival are reported as mean values with standard errors (SE). Fisher’s least significant difference (LSD) test was used to separate means (P < 0.01). The software SPSS v17.0 (IBM Inc., Chicago) was used for data analyses.

## Results

### Nymphiposition, nymphal duration, and survival

**Figure 1:**
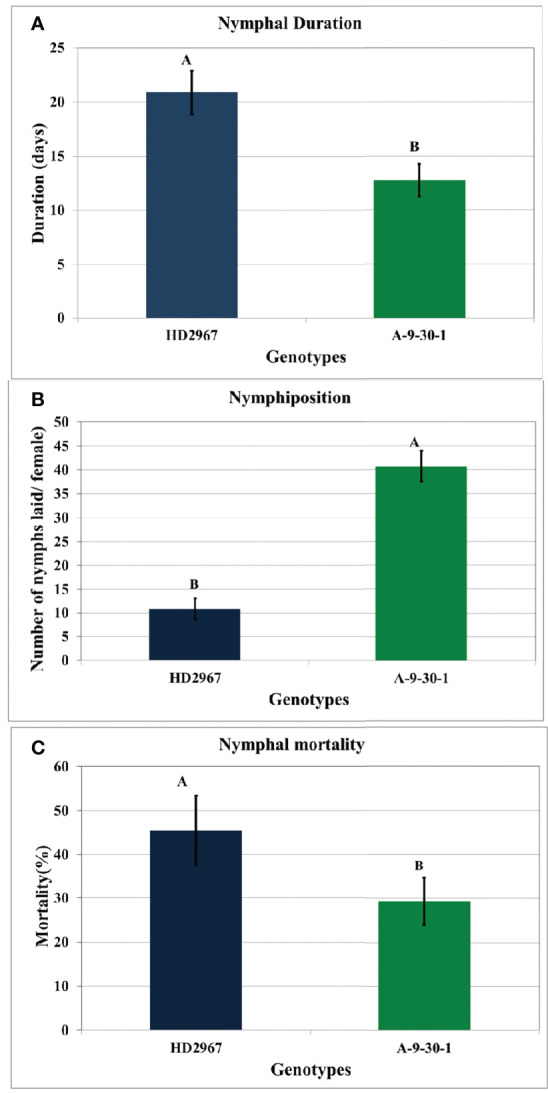
Biological parameters **(A)** nymphal duration (days) **(B)** nymphiposition (nymphs/female) **(C)** nymphal mortality (%) of *R. maidis* recorded on susceptible durum wheat genotype, A-9-30-1 and tolerant aestivum wheat genotype, HD2967. Means indicated by the different letters are significantly different from each other (ANOVA; LSD Test; P ≤ 0.05). Standard errors of means are indicated by error bars.

There was a significant difference in the nymphiposition of *R. maidis* recorded on wheat susceptible genotype A-9-30-1 and tolerant genotype HD2967. The nymphiposition (fecundity) of *R. maidis* was higher (40.72 ± 0.54 nymphs) on A-9-30-1 as compared to HD2967 (10.81 ± 0.75 nymphs). There were also significant differences in nymphal duration and survival between the two genotypes studied. The nymphal duration of 12.75 days was recorded on A-9-30-1 as compared to 20.89 days on genotype HD2967. The nymphal mortality rate on genotype HD2967 was higher (45.79%) than on A-9-30-1 (29.36%) (**Figure 1**).

### RNA-seq data summary

By using Illumina sequencing technology, a total of 59,691,508 (8.99 Gb), 56,052,968 (8.43 Gb), 47,363,964 (7.13 Gb), and 56,403,641 (8.49 Gb) numbers of reads were generated for A-9-30-1 infested, A-9-30-1 uninfested, HD2967 infested, and HD2967 uninfested, respectively. These high-quality reads were mapped onto the reference genome through TopHat v2.1.1 with default parameters. The mapping statistics for A-9-30-1 infested leaf and A-9-30-1 uninfested leaf were 47% and 87.8%, respectively. However, it was 66.8% and 89.3% for HD2967 infested leaf and HD2967 uninfested leaf, respectively (**Table 1**).

**Table 1:** High quality read statistics of susceptible genotype *Triticum durum* A-9-30-1 line and tolerant genotype *Triticum aestivum* HD 2967.

Sr. No.	Sample	Number of filtered reads	Total number of bases	Data in Gb	Mapping %
**1.**	A-9-30-1 infested leaf	59,691,508	8,987,046,576	8.99	47.0
**2.**	A-9-30-1uninfested leaf	56,052,968	8,434,060,281	8.43	87.8
**3.**	HD 2967 infested leaf	47,363,964	7,129,315,520	7.13	66.8
**4.**	HD 2967 uninfested leaf	56,403,641	8,490,579,497	8.49	89.3

### Identification of differentially expressed genes

**Figure 2:**
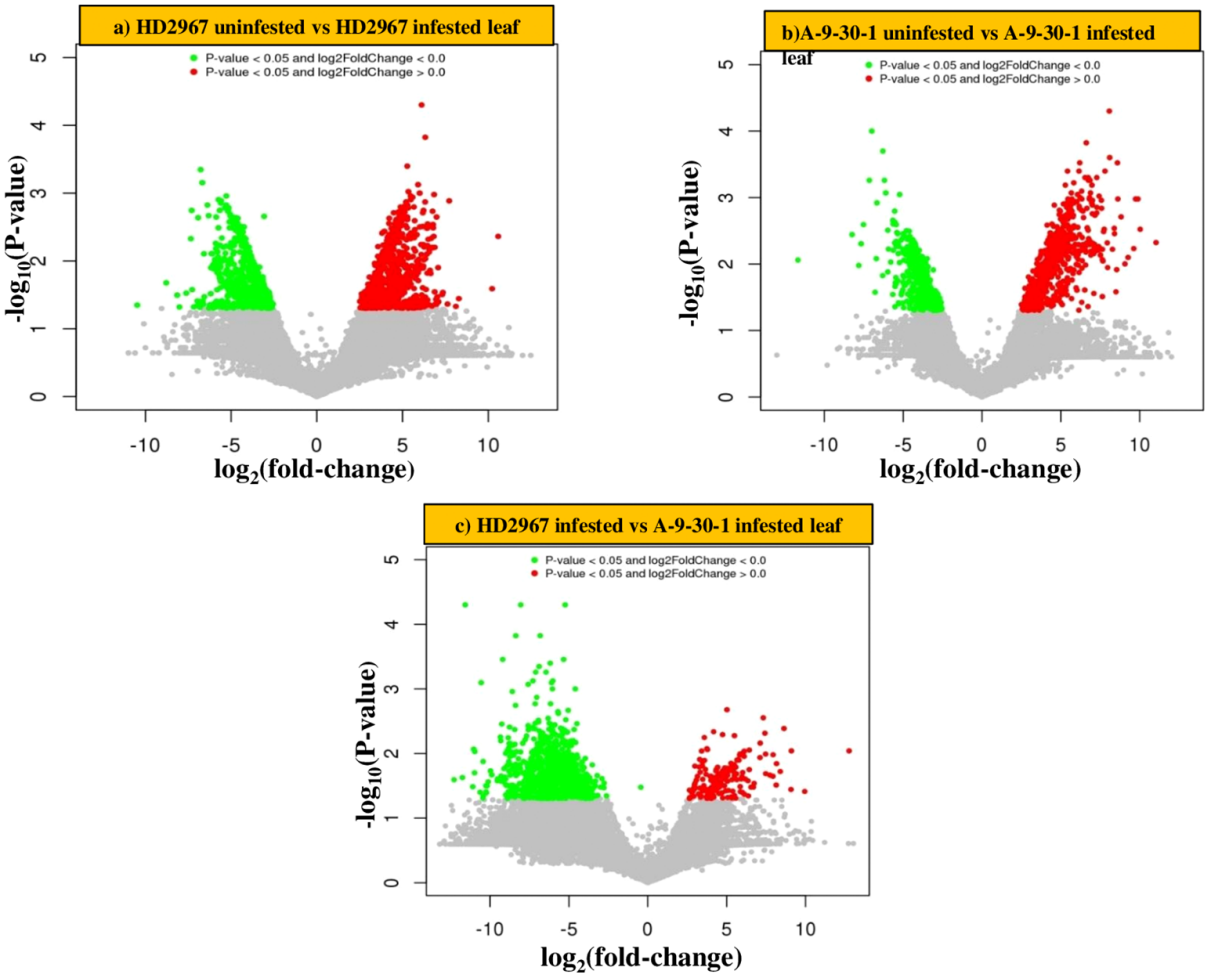
Differentially expressed genes (DEGs) revealing Volcano plots **(A)** HD2967 uninfested leaf vs HD2967 infested leaf after 48 h **(B)** A-9-30-1 uninfested leaf vs A-9-30-1 infested leaf after 48 h **(C)** HD2967 infested leaf vs A-9-30-1 infested leaf after 48 h of *R. maidis* feeding. The red block on the right side of zero represents the upregulated genes, whereas the green block on the left side of zero represents significant downregulated genes. The Y-axis represents the negative log of P-value (*p* value <=0.05) of the performed statistical test, where data points with low p-values (highly significant) appearing towards the top of the plot. The grey block shows the non-differentially expressed genes.

Transcriptome analysis of two selected genotypes, A-9-30-1 and HD2967 revealed a significant difference in gene expression after aphid attack. In combination 1 (tolerant genotype HD2967 infested vs uninfested), 1183 and 959 genes were significantly upregulated and downregulated, respectively. Similarly in combination 2 (susceptible A-9-30-1 infested vs uninfested), a reduction in the number of upregulated (1055) and downregulated genes (765) was observed. On the other side, comparative analysis of susceptible and tolerant genotypes without aphid infestation (combination 3), it was noticed that 353 and 1175 genes were upregulated and downregulated, respectively. Further in combination 4 when both genotypes were infested with aphids, only 212 genes were significantly upregulated and 1009 genes were significantly downregulated. (**Table 2**). The proprietary R script was used to depict the graphical representation and distribution of differentially expressed genes in control as well as treated samples. The ‘volcano plot’ arranges expressed genes along dimensions of biological as well as statistical significance (**Figure 2**).

**Table 2:** DGE summary of the four combinations.

Sr No.	Combination	Combination 1*(HD2967 infested leaf vs HD2967 uninfested leaf control)*	Combination 2 *(A-9-30-1 infested leaf vs A-9-30-1 uninfested leaf control)*	Combination 3 *(A-9-30-1 uninfested leaf vs HD2967 uninfested leaf control)*	Combination 4 *(HD2967 infested leaf vs A-9-30-1 infested leaf (control))*
1.	Upregulated	1183	1055	3153	212
2.	Downregulated	959	765	1175	1009

### Gene ontology enrichment of differentially expressed genes

**Figure 3:**
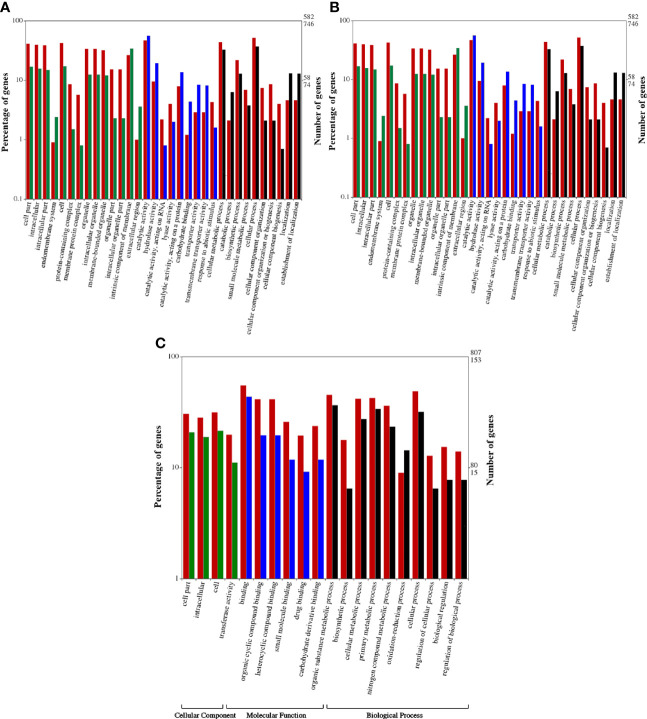
**(A)** Gene ontological analysis of tolerant aestivum wheat genotype, HD2967 uninfested leaf in comparison to HD2967 infested leaf after 48 h of *R. maidis* feeding. Red bars indicate downregulated genes whereas green, blue and black bars indicate upregulated genes of cellular, molecular and biological processes, respectively. **(B)** Gene ontological analysis of susceptible durum wheat genotype, A -9-30-1 uninfested leaf in comparison to A-9-30-1 infested leaf after 48 h of *R. maidis* feeding. Red bars indicate downregulated genes whereas green, blue and black bars indicate upregulated genes of cellular, molecular and biological processes, respectively. **(C)** Gene ontological analysis of tolerant aestivum wheat genotype, HD2967 infested leaf in comparison to susceptible durum wheat genotype, A -9-30-1 infested leaf after 48 h of *R. maidis* feeding. Red bars indicate downregulated genes whereas green, blue and black bars indicate upregulated genes of cellular, molecular and biological processes, respectively.

The DEGs obtained were subjected to GO enrichment analysis for the identification of the main biological functions of DEGs in wheat under biotic stress conditions (Young et al., 2010). All DEGs were classified into three categories, i.e. cellular components (CC), molecular function (MF), and biological processes (BP) (Ashburner et al., 2000; Sharma et al., 2019) **Figures 3A–C**). Annotation of DEGs of HD2967 (infested vs uninfested) revealed that a total of 4,364 GO terms corresponding to 2062 molecular functions, 1323 biological processes, and 979 cellular components were assigned. “Transferase activity,” “catalytic activity (acting on a protein),” “organic cyclic compound binding,” and “heterocyclic compound binding activity” were the most dominant GO terms in the MF category, while “nitrogen compound metabolic process,” “regulation of the metabolic process,” “oxidation-reduction process,” “cell communication,” and “cellular component organization” were the most dominant GO terms in the BP category. However, in the case of CC, most of the DEGs were classified into “cell part”, “intracellular part”, “cell”, and “protein-containing complex”, followed by “membrane-bounded organelle”. However, the annotation of DEGs of A-9-30-1 (infested vs uninfested) showed a total of 3,255 GO terms, which contained 1531 molecular functions, 1012 biological processes, and 712 cellular components. The dominant terms in the MF category were “catalytic activity, acting on RNA”, “lyase activity”, “carbohydrate binding” and transporter activity. In the case of BP, “cellular metabolic processes”, “catabolic processes”, “biosynthetic process” and “small molecule metabolic process”, whereas, “cell part”, “intracellular part”, “endomembrane system”, and “protein-containing complex” were the most dominant GO terms (**Figures 3A–C**).

On a comparison of infested HD2967 and infested A-9-30-1, a total of 621 GO terms was identified which corresponds to 257 MF, 193 BP, and 171 CC. The GO assignment for MF involves “binding”, “organic cyclic compound binding”, “heterocyclic compound binding” and “small molecule binding” whereas, BP involved “biosynthetic process”, “cellular metabolic process”, “nitrogen compound metabolic process” and oxidation-reduction process. In the case of CC, the dominant GO terms include “cell part”, “intracellular” and “transferase activity” (**Figures 3A–C**).

### Kyoto encyclopedia of genes and genomes under stress condition

The KEGG pathway analysis was performed for the understanding of active biological pathways during aphid attack in wheat (**Supplementary information: Excel-Sheets** showing all pathways). The analysis revealed that out of 127 KEGG pathways, 25 were significantly active enriched at a p-value <0.05, covering five main KEGG categories such as metabolism, genetic information processing, environmental information processing, cellular processes, and organismal systems. The KEGG pathways associated with DEGs under aphid attack conditions were widely associated with metabolism, biosynthesis of secondary metabolites, and plant hormone signal transduction. The genes involved in these 25 pathways were upregulated in HD2967 as well as in A-9-30-1 after aphid attack (**Table 3**).

**Table 3:** Classification of Kyoto Encyclopedia of Genes and Genomes (KEGG) Pathway.

Sr. No.	Pathways	*Triticum durum*		*Triticum aestivum*	
		*A-9-30-1 infested leaf*	*A-9-30-1 uninfested leaf*	*HD2967 uninfested leaf*	*HD2967 infested leaf*
**Metabolism**					
1.	Carbohydrate metabolism	927	1005	1195	1173
2.	Energy metabolism	599	642	738	710
3.	Lipid metabolism	529	568	715	701
4.	Nucleotide metabolism	276	332	400	373
5.	Amino acid metabolism	656	678	835	857
6.	Metabolism of other amino acids	293	300	373	378
7.	Glycan biosynthesis and metabolism	184	205	260	249
8.	Metabolism of cofactors and vitamins	471	467	575	549
9.	Metabolism of terpenoids and polyketides	195	209	258	243
10.	Biosynthesis of other secondary metabolites	209	219	282	298
11.	Xenobiotics biodegradation and metabolism	196	193	244	253
12.	Enzyme families	1	1	1	0
**Genetic information processing**					
1.	Transcription	434	480	586	561
2.	Translation	1095	1209	1462	1427
3.	Folding, sorting and degradation	850	908	1104	1071
4.	Replication and repair	147	205	256	222
**Environmental information processing**					
1.	Membrane transport	46	59	69	67
2.	Signal transduction	1033	1103	1321	1288
3.	Signaling molecule and interaction	4	4	6	6
**Cellular processes**					
1.	Transport and catabolism	791	814	968	961
2.	Cell growth and death	455	509	609	572
3.	Cellular community-eukaryotes	140	152	175	172
4.	Cellular community-prokaryotes	100	118	134	137
5.	Cell motility	82	87	104	100
**Organismal systems**					
1.	Environmental adaptation	447	459	566	534

### Transcripts of photosynthesis, starch, sucrose, and nitrogen metabolism

*R. maidis* attack on HD2967 and A-9-30-1 wheat showed its negative effect on the photosynthesis process in which a large number of genes associated with light-harvesting and photosystem, i.e. chlorophyll a-b binding proteins, photosystem I and II, and ferrochelatase were significantly downregulated (**Table 4**). The expression levels of transcripts of carbonic anhydrase and ribulose bisphosphate carboxylase oxygenase (RuBisCO) genes were also significantly downregulated, as these genes are mainly involved in the Calvin cycle. Transcription profiles for sucrose and starch metabolism were also analyzed for a few genes, which showed that one trehalose-6-phosphate synthase gene, one sucrose synthase-3 gene, and six beta-glucosidase genes were also significantly downregulated during the attack of the *R. maidis*. However, the sucrose-phosphate gene is upregulated during the attack of *R. maidis* on HD2967 wheat leaves. The level of nitrate reductase transcripts was strongly downregulated by *R. maidis* in both, i.e., in HD2967 and A-9-30-1, whereas glutamate dehydrogenase transcript levels were significantly upregulated in HD2967 wheat leaves infested with *R. maidis* (**Table 4**).

**Table 4:** Differentially expressed genes (DEGs) associated with primary plant metabolism.

Pathways	Gene description	Gene ID	*Triticum durum*		*Triticum aestivum*	
			Log_2_ FC	P-value	Log_2_ FC	P-value
**Photosynthesis**	Chlorophyll a-b binding protein	TraesCS5B02G462800	-6.8352	1.98E-05	-6.982	1.79E-02
		TraesCS5A02G454200	-6.031	5.13E-06	-5.931	5.20E-05
		TraesCS5B02G462900	-6.839	3.60E-09	-6.521	3.45E-03
		TraesCS1A02G403800	-6.985	5.19E-11	-4.234	1.02E-05
		TraesCS6A02G094200	-7.317	3.20E-16	-3.951	3.04E-02
		TraesCS6A02G094500	-7.052	3.53E-27	-5.732	1.25E-04
		TraesCS5B02G463000	-7.389	4.42E-10	-5.632	5.90E-03
		TraesCS6A02G094600	-8.35	5.71E-05	-5.20	3.30E-06
	Ferrochelatase	TraesCS1A02G135700	-5.098	2.65E-03	-4.512	5.53E-02
	Photosystem I proteins	TraesCS1B02G420100	-6.922	2.23E-05	-6.305	2.15E-04
		TraesCS2A02G252600	-4.51	4.412E-02	-4.207	5.23E-02
		TraesCS2B02G272300	-5.032	2.290E-05	-7.32	4.40E-04
		TraesCS1A02G392000	-6.162	2.12E-03	-6.992	2.30E-03
		TraesCS5A02G256900	-4.312	3.10E-09	-4.412	3.10E-04
	Photosystem II proteins	TraesCS4A02G355600	-4.369	4.65E-05	-4.822	5.90E-02
		TraesCS5A02G386400	-3.045	3.35E-03	-5.002	1.01E-05
		TraesCS7B02G215000	-4.093	2.47E-06	-4.992	1.48E-03
		TraesCS7A02G314100	-6.06	4.82E-02	-5.102	2.195E-03
		TraesCS3B02G344200	-7.128	1.26E-02	-4.182	3.862E-06
		TraesCS7A02G314100	-7.412	0.552E-03	-4.061	1.63E-04
	Photosynthetic NDH	TraesCS6A02G308100	-3.68	1.723E-04	-4.379	2.62E-03
	Carbonic anhydrase	TraesCS3A02G230000	-3.852	1.652E-05	-3.110	4.19E-02
		TraesCS3B02G259300	-2.977	2.99E-03	-3.273	3.46E-04
**Sucrose and starch metabolism**	Trehalose-6-phosphate synthase	TraesCS1A02G339300	-3.793	1.49E-04	-4.018	0.89E-04
	sucrose synthase 3	TraesCS2A02G168200	-3.917	4.455E-03	-3.173	3.78E-03
	Sucrose-phosphatase	TraesCS1B02G107600	-2.901	4.051E-02	2.843	3.25E-02
	Beta-glucosidase	TraesCS2B02G401500	-3.914	1.585E-03	-2.974	4.55E-05
**Nitrogen metabolism**	Nitrate reductase	TraesCS6B02G356800	Induced	0.93E-05	Induced	1.571E-04
		TraesCS6A02G326200	Induced	3.77E-02	Induced	0.921E-03
	Glutamine synthetase	TraesCS2A02G500400	-3.370	0.47E-05	-3.389	0.85E-03
		TraesCS2B02G528300	-3.291	0.04E-05	-4.335	1.69E-03
		TraesCS6B02G327500	-1.891	1.68E-08	-3.760	0.71E-03
	Glutamate dehydrogenase	TraesCS2B02G409300	-4.176	2.71E-03	2.889	0.74E04
		TraesCS2A02G389900	-3.035	2.21E-04	4.057	4.90E02
	Cysteine synthase	TraesCS3A02G338600	-3.084	4.51E-02	-4.507	4.41E-02
		TraesCS3B02G370200	-3.003	3.82E-02	-2.828	2.39E-03
		TraesCS4A02G401600	-3.267	2.83E-06	-2.992	1.85E-02
		TraesCS6B02G217200	-3.593	3.38E-03	-3.354	2.63E-04

### Plant defense response against *R. maidis* attack

Characterization of genes involved in jasmonic acid (JA), salicylic acid (SA), and ethylene (ET) defense pathways were used to estimate plant defense response to *R. maidis* attack (**Table 5**). It was found that in SA biosynthesis pathway, 15 phenylalanine ammonia-lyase (PAL) genes and 9 pathogenesis-related genes (PR) were significantly upregulated in response to *R. maidis* feeding in both, i.e., in *T. durum* (A-9-30-1) and *T. aestivum* (HD2967). When the expression of these PAL genes and PR genes was compared, it was observed that it was significantly lower in *T. durum* (A-9-30-1) than their expression in *T. aestivum* (HD2967). Furthermore, when *R. maidis* attacked *T. durum* (A-9-30-1) and *T. aestivum* (HD2967), the expression level of two AOS genes varied from 1.08 to 2.07-fold in A-9-30-1 and 4.14 to 4.95-fold in HD2967. The expression of three LOX expressions varied from 3.13 to 5.83-fold in A-9-30-1 and 4.10 to 6.02-fold in HD2967, indicating its significant up-regulation in JA biosynthesis. The expression level of two PI genes (JA-defense responsive genes) was found to be higher in the tolerant genotype, *T. aestivum* (HD2967), as compared to the susceptible genotype, *T. durum* (A-9-30-1). It varied from 3.12 to 4.69-fold in A-9-30-1 and 3.14 to 5.71-fold in HD2967. Furthermore, it was found that in the ET signaling pathway, one 1-aminocyclopropane-1-carboxylate synthase (ACS) gene, four 1-aminocyclopropane-1-carboxylate oxidase 1 (ACO), three genes belonging to ET-responsive transcription factors and ET-insensitive protein genes were all significantly upregulated in response to *R. maidis* feeding in A-9-30-1 and HD2967 (**Table 5**).

**Table 5:** Differentially expressed genes (DEGs) involved in salicylic acid (SA), jasmonic acid (JA), and ethylene (ET) dependent defense pathways.

Pathways	Gene description	Gene ID	*Triticum durum*		*Triticum aestivum*	
			Log_2_ FC	P-value	Log_2_ FC	P-value
SA-defense pathway	Phenylalanine ammonia-lyase (PAL)	TraesCS1A02G037700	2.33	1.10E-04	4.33	2.135E-02
		TraesCS1B02G122800	3.99	0.63E-02	5.185	2.25E-03
		TraesCS2A02G196400	1.83	1.34E-02	3.48	2.96E-02
		TraesCS2A02G196700	0.32	3.39E-03	3.54	2.33E-03
		TraesCS2A02G381000	4.88	0.82E-03	6.81	1.05E-03
		TraesCS2A02G381100	3.13	3.66E-02	4.83	0.76E-02
		TraesCS2B02G224300	3.21	2.55E-02	3.68	3.17E-04
		TraesCS2B02G398100	5.46	0.85E-03	6.77	4.15E-05
		TraesCS2B02G398200	1.29	3.10E-03	3.14	4.56E-02
		TraesCS2B02G398400	3.04	4.28E-02	3.98	1.06E-03
		TraesCS4A02G401300	0.65	1.19E-02	3.52	2.21E-04
		TraesCS5B02G468300	0.59	0.53E02	3.07	4.35E-03
		TraesCS5B02G468400	0.19	0.04E-06	3.91	2.44E-03
		TraesCS6A02G222700	1.13	4.09E-03	6.34	1.05E-04
		TraesCS6B02G258600	0.81	0.89E-03	7.95	0.48E-03
	Pathogenesis-related protein (PR protein)	TraesCS1A02G355300	1.31	0.97E-02	4.29	3.90E-05
		TraesCS1B02G366300	2.68	2.85E-04	4.43	1.15E-02
		TraesCS3A02G480400	1.32	4.60E-04	3.04	4.31E-04
		TraesCS3B02G525000	2.75	0.30E-06	3.47	2.25E-03
		TraesCS5A02G018200	3.31	3.75E-03	5.60	1.55E-04
		TraesCS5A02G183300	2.09	0.01E-03	5.03	0.42E-05
		TraesCS5A02G439800	5.11	1.95E-04	5.26	0.48E-03
		TraesCS5B02G181500	3.05	0.44E-02	3.82	1.60E-03
		TraesCS5B02G442700	0.01	1.34E-03	3.97	1.01E-03
JA-defense pathway	Allene oxide synthase (AOS)	TraesCS4A02G061900	2.07	0.96E-03	4.95	1.23E-03
		TraesCS4B02G237600	1.08	1.38E-02	4.14	1.57E-04
	Lipoxygenase (LOX)	TraesCS4B02G037700	3.13	1.96E-02	4.10	0.75E-04
		TraesCS4B02G037800	5.83	0.57E-03	6.02	1.15E-03
		TraesCS4B02G037900	4.30	3.48E-02	5.49	1.25E-03
	Proteinase inhibitors (PIs)	TraesCS3A02G046100	4.69	0.41E-03	5.71	0.55E-03
		TraesCS3B02G038700	3.12	2.75E-03	3.14	3.66E-03
ET-signaling pathway	1-aminocyclopropane-1-carboxylate synthase (ACS)	TraesCS2B02G414800	1.63	0.59E-02	3.00	4.62E-02
	1-aminocyclopropane-1-carboxylate oxidase 1 (ACO)	TraesCS6B02G355900	2.89	4.2E-02	2.95	1.57E-03
		TraesCS6A02G325700	2.00	0.72E-03	4.58	0.50E-02
		TraesCS6B02G356200	1.41	0.35E-03	6.41	0.51E-03
		TraesCS4A02G109200	3.35	2.95E-05	4.08	0.72E-03
	ET-responsive transcription factors	TraesCS6B02G281000	2.80	0.51E-03	3.23	3.43E-04
		TraesCS4A02G326400	2.41	0.61E-03	3.01	4.24E-03
		TraesCS1B02G282300	2.98	4.84E-02	4.46	0.66E-02
	ET-insensitive protein	TraesCS5A02G547500	3.08	4.33E-03	5.23	0.61E-03
		TraesCS4A02G275600	2.73	2.66E-04	3.00	4.46E-02
		TraesCS7B02G145400	3.02	0.5E-05	3.07	4.27E-05

**Figure 4:**
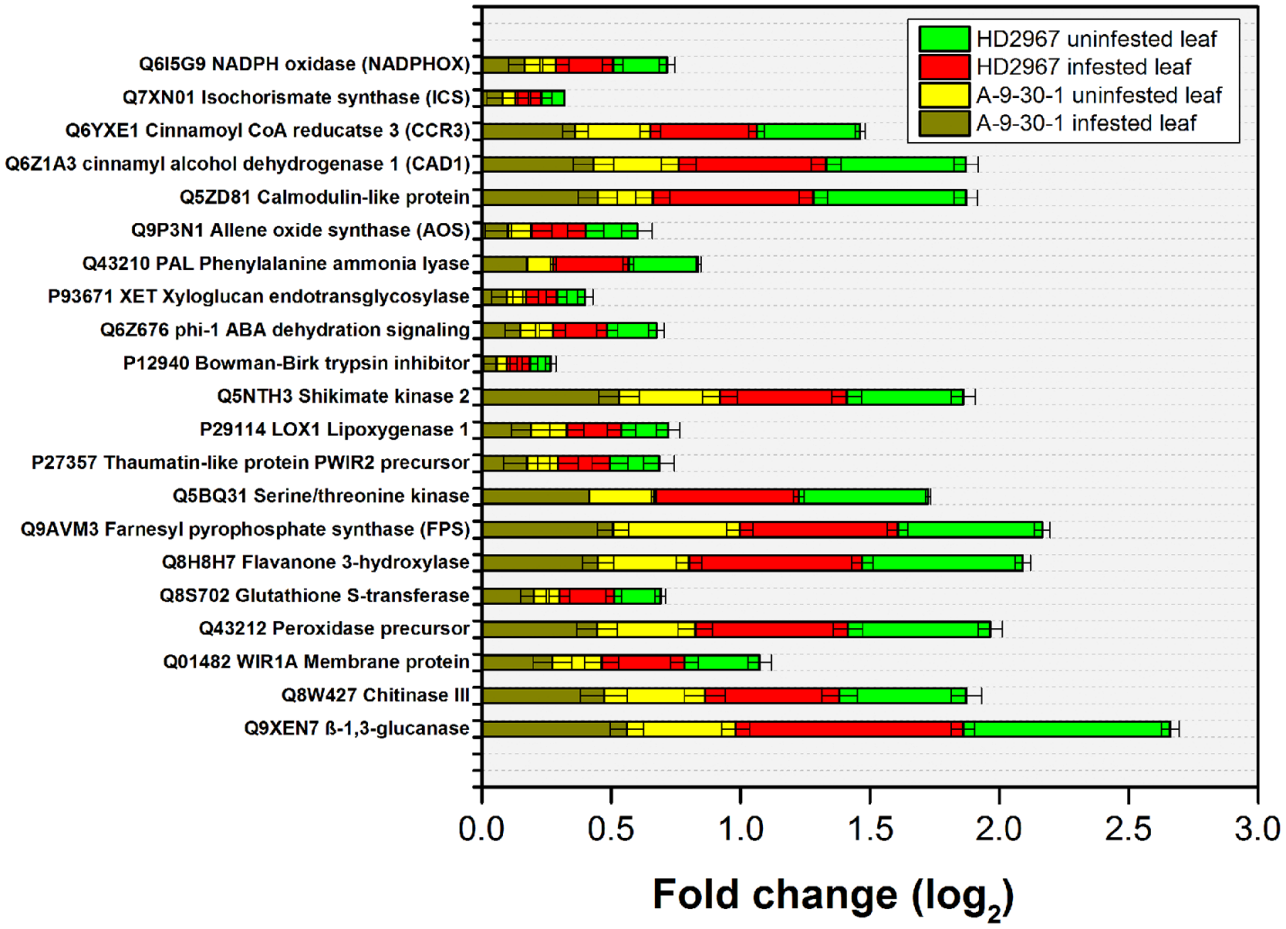
Spatiotemporal real-time PCR expression analysis of defense-related genes in wheat before and after attack of aphid.

A large number of pathogenesis/defense-related genes were found to be differentially expressed under aphid-attack conditions. In total, 21 important pathogenesis/defense-related genes were selected for expression analysis in control as well as infested samples of wheat, i.e., *T. durum* and *T. aestivum* against *R. maidis* (**Figure 4**). It was found that a serine/threonine kinase DEG showed upregulated expression in infested HD2967 whereas downregulated expression was in uninfested A-9-30-1. Similarly, a gene encoding signaling-associated proteins and other stress tolerance were observed, namely “Q5ZD81 Calmodulin-like protein”. It showed increased expression in infested HD2967 and decreased expression in uninfested A-9-30-1. The DEGs related to plant cell protection from oxidative damage by ROS scavenging, such as “Q8S702 Glutathione S-transferase” and “Q43212 Peroxidase precursor” showed their increased expression in infested HD2967, whereas downregulated expression was seen in uninfested A-9-30-1. Overall, Q9XEN7 β-1, 3-glucanase had the highest expression in the infested *T. aestivum* and *T. durum* whereas P12940 Bowman-Birk trypsin inhibitor had the lowest expression in the infested and uninfested *T. aestivum* and *T. durum* (**Figure 4**). 

## Discussion

Plants have the ability to evolve various morphological and physiological mechanisms in response to stress conditions, which helps in countering their negative impacts on fitness (Karban, 2020). Aphids evolve a complex relationship with their host by secreting signaling compounds (‘elicitors’) that directly affect gene expression and metabolism of plants in a way that subverts normal plant defense responses and improves phloem content quality as food for plant fitness (Liu et al., 2020). In the present research, we examined the plant defense responses in susceptible genotypes of *T. durum* i.e. A-9-30-1) and tolerant *T. aestivum* genotypes, i.e., HD2967 following infestation by *R. maidis*. These genotypes were characterized as susceptible or tolerant based on screening of these genotypes against *R. maidis* in the field and under glasshouse conditions. Host plant defense response in the form of nymphiposition, nymphal duration, and survival presented in this study clearly showed a difference in their resistance response against *R. maidis*. The nymphiposition (fecundity) of *R. maidis* was higher on A-9-30-1, however nymphal duration and survival were lower as compared to HD2967. These significant differences in nymphiposition, nymphal duration, and survival between the two genotypes clearly indicated that genotype HD2967 had a higher resistance response against *R. maidis* than A-9-30-1. Previous studies conducted on the screening of identification of aphid-resistant genotypes in wheat crops had reported promising aphid resistance response in HD2967 (Kaur et al., 2017; Devrani et al., 2018; Sharma & Singh, 2022).

Further, we hypothesized that during aphid-host interaction, transcriptomic changes occur that are associated with the plant’s response to aphid infestation. Transcriptomic changes were studied by comparing the transcriptome profiles and by determining the changes in gene expression levels of two contrasting wheat genotypes. The study revealed that the response of A-9-30-1-infested wheat leaf to *R. maidis* is different from that of HD2967-infested wheat leaf to *R. maidis*. The results indicated a significant number of differentially expressed genes between two chosen genotypes, A-9-30-1, and HD2967. Transcriptomic variations by comparing transcriptome profiles have been previously reported and differences in the gene expression among contrasting genotypes for biotic (Batyrshina et al., 2020; Correa et al., 2020) and abiotic stress traits (Hu et al., 2018) were reported.

Plant pathogenesis-related (PR) genes play an important role in the defense mechanisms against biotic factors in plants (Montenegro et al., 2017; Li et al., 2021). Overexpression of these genes increases resistance against various pathogens in different crops (Manghwar et al., 2018). By comparing the transcriptome profiles of two contrasting genotypes, it was found that despite subjecting plants to the same stress conditions, the expression of genes was significantly different between the tolerant and the susceptible genotype. A significantly higher number of DEGs, a total of 212 genes, were significantly upregulated in HD2967 after aphid attack when it was compared with an A-9-30-1 infested leaf. These defense-related genes might play a role in insect resistance against *R. maidis*. Further evaluation of gene responses to *R. maidis* attack at the molecular level revealed that the photosynthesis, sucrose and starch metabolism, and nitrogen metabolism genes were downregulated in both genotypes. Similar studies revealed that after 48 hours of infestation, *Schizaphis graminum* attack caused serious chlorosis and chlorophyll loss in genotypes Beijing 837 and Zhongmai 175 (Wang et al., 2017; Zhang et al., 2019). Transcriptomic and metabolic analysis of wild and domesticated wheat genotypes revealed differences between the abundance of defense mechanisms in the wild and domesticated plants were observed in which wild emmer possesses high physical defenses while the domesticated wheat genotypes have high chemical defenses (Batyrshina et al., 2020). Morpho-histological and gene expression analyses of two wheat cultivars (BRS Timbaúva, resistant, and Embrapa 16, susceptible) challenged and unchallenged by *R. padi* indicated that green leaf volatiles was involved in the aphid resistance and trichomes were more abundant and larger in the resistant cultivar. Further, the lipoxygenase-encoding gene was downregulated in the susceptible cultivar and basal expression remained level in the resistant cultivar, however, the expression of resistance-related proteins was induced in the resistant but not in the susceptible cultivar (Correa et al., 2020). According to GO analysis, differentially expressed genes of A-9-30-1 and HD2967 were classified into three main domains, such as biological processes, cellular components, and molecular function. The results obtained from the present study showed that metabolic processes and cellular processes had the highest number of DEGs during host-aphid interaction. The obtained results of GO analysis support the hypothesis that plant interaction with insect pests causes changes in their primary (plant growth and development) and secondary (induction of defense program) metabolisms. When plants become infested with insect pests, they begin to expend more energy on defense gene activation than on growth, development, reproduction, and cellular maintenance (Carroll and Moore, 2018). From this study, it was concluded that *R. maidis* feeding caused chlorosis, which strongly downregulated plant photosynthesis, starch, sucrose, and nitrogen metabolism. Further, it was found that the DEGs metabolic processes are related to the defense mechanisms of *R. maidis* as well as plant-pest interactions.

KEGG pathway analysis revealed that the most DEGs were involved in carbohydrate metabolism, energy metabolism, lipid metabolism, nucleotide metabolism, amino acid metabolism, glycan biosynthesis and metabolism, metabolism of cofactors and vitamins, metabolism of terpenoids and polyketides, biosynthesis of other secondary metabolites, xenobiotic biodegradation and metabolism, enzyme families, transcription, translation, folding, sorting and degradation, replication and repair, membrane transport, signal transduction, signaling molecule and interaction, transport and catabolism, cell growth and death, cellular community-eukaryotes, cellular community-prokaryotes, cell motility, and environmental adaptation were upregulated in the tolerant genotype as compared to susceptible. The results obtained in the present study were comparable with the results obtained in previous studies on wheat after *S. graminum* and *S. avenae* attacks (Zhang et al., 2019).

Herbivorous insect attacks on plants induce modulated defense responses such as up-regulation of plant hormones including jasmonic acid (JA), ethylene (ET), salicylic acid (SA), gibberellic acid (GA), and abscisic acid (ABA) (Bari and Jones, 2009; Mertens et al., 2021). Plants limit herbivory by activating toxic secondary metabolites and defensive protein production such as protease inhibitors, glucosinolates, lectins, and 2, 4-dihydroxy-7-methoxy-2H-1,4-benzoxazin-3(4H)-one (DIMBOA) (Morkunas et al., 2011). However, insects also have various enzymes that degrade toxins and facilitate the host’s adaptation to adverse environmental conditions (Rong et al., 2016). Whiteflies and aphids are piercing-sucking insects and these insect pests activate the SA-mediated defense signaling pathway in wheat (Züst and Agrawal, 2016; Wang et al., 2017). Previous studies have reported that the genes of SA and JA defense pathways such as PAL PIs, PR1, and LOX were significantly upregulated against aphid attack in wheat (Moran and Thompson, 2001; Zhu-Salzman et al., 2004; Reddy et al., 2013). Similar results were found in the present study that the *R. maidis* attack significantly increased the gene expression of the JA, SA, and ET signaling pathways. Although both genotypes induced the expression of SA, JA, and ET genes, the expression of these genes was greater in HD2967 as compared to A-9-30-1. Zhang et al. (2019) demonstrated that the PR gene expression was significantly greater after *S. graminum* feeding on wheat. It was also observed that the activation of plant defense responses was closely related to the plant species, infestation time, and aphid density (Stewart et al., 2016: Hodge et al., 2019). Overall, our finding provides a genome-wide gene expression profiling for wheat plants infested with *R. maidis*, which helps to elucidate the regulatory resistance mechanisms in wheat against aphids.

## Conclusions

The transcriptomic profiling of two wheat genotypes revealed differences among the responses of wheat to feeding by aphid *R. maidis* on susceptible genotype, A-9-30-1 and tolerant genotype, HD2967. Higher nymphiposition and lower nymphal duration and nymphal mortality of *R. maidis* were observed on the susceptible genotype, A-9-30-1 as compared to the tolerant genotype, HD2967. *R. maidis* feeding caused chlorosis, which strongly downregulated plant photosynthesis, starch, sucrose, and nitrogen metabolism. Comparatively, a higher number of DEGs were significantly upregulated after aphid attack in tolerant genotype HD2967 as compared to susceptible genotype, A-9-30-1. As per GO analysis, metabolic processes and cellular processes had the highest number of DEGs during host-aphid interaction. From this study, it was concluded that pathogenesis/defense-related genes do express in both genotypes but their expression levels increased after the aphid attack and varied among the two selected genotypes.

The findings of the study provide new insights into the defense adaptations of wheat plants against aphids. Overall, our results provide an important foundation for further understandingthe aphid resistance mechanisms of selected resistant genotypes that will help us to breed*R. maidis* resistant wheat genotypes.

## Data availability statement

The datasets presented in this study can be found in online repositories. The names of the repository/repositories and accession number(s) can be found below: https://www.ncbi.nlm.nih.gov/, SAMN09463019 https://www.ncbi.nlm.nih.gov/, SAMN09463022.

## Ethics statement

The animal study was reviewed and approved by ICAR.

## Author contributions

PJ, PK & SS: conceptualized and conducted the study and wrote the manuscript MN & DK: Maintained insect culture and did RNA extractions SaK, CM, SuK and GS: Assisted in planning of the study, provided plant material, and helped in reviewing the manuscript. All authors contributed to the article and approved the submitted version.

## Conflict of interest

The authors declare that the research was conducted in the absence of any commercial or financial relationships that could be construed as a potential conflict of interest.

## Publisher’s note

All claims expressed in this article are solely those of the authors and do not necessarily represent those of their affiliated organizations, or those of the publisher, the editors and the reviewers. Any product that may be evaluated in this article, or claim that may be made by its manufacturer, is not guaranteed or endorsed by the publisher.

## Supplementary Material

The Supplementary Material for this article can be foundonline at: https://www.frontiersin.org/articles/10.3389/fpls.2022.989365/full#supplementary-material
