# Supplementary material for: Comparative transcriptome analysis of wheat in response to corn leaf aphid, Rhopalosiphum maidis F. infestation
Source: Front Plant Sci. 2022 Nov 24;13:989365. doi: 10.3389/fpls.2022.989365 (PMC9730506; doi:10.3389/fpls.2022.989365)
Supplement: Supplementary file 1 [file Table_1.docx]

**Supplementary Information**

**Table 1: Detail of primers of defense-related proteins (PRs) of wheat used for the present study**

| **Sr. No.** | **Name of genes** | **Primer sequence (5′–3′)** | **Base pair** |
| --- | --- | --- | --- |
| 1. | Q9XEN7 β-1,3-glucanase | **F:**CTCTTCAACCCGGACAAATC  **R:**TGAAGAATTTGGGCGTTTTC | 20  20 |
| 2. | Q8W427 Chitinase III | **F:** CGACAACCTGGACTGCTACA  **R:** ATGGATCGCACCATTATTCG | 20  20 |
| 3. | Q01482 WIR1A Membrane protein | **F:** CTCCTGCAGATCGCTCTCTT  **R:** CCGGTGGTCTACATCCGTAA | 20  20 |
| 4. | Q43212 Peroxidase precursor | **F:** AACACTGTCCGGAACTTTGC  **R:** TGTCGTGCTGGCTAGTATGC | 20  20 |
| 5. | Q8S702 Glutathione S-transferase | **F:** CCTCAGGGACTGCTCTAACG  **R:** GTCCAACGATCCGAAGTTGT | 20  20 |
| 6. | Q8H8H7 Flavanone 3-hydroxylase | **F:** TACCGCAGCTACACCTACGA  **R:** TGAGTAATGCTGCGTCGTG | 20  19 |
| 7. | Q9AVM3 Farnesyl pyrophosphate synthase (FPS) | **F:**CATCATTGACATGTCCTGAAAA  **R:** GGGCTTGCAGTAAGCAAAAA | 22  20 |
| 8. | Q5BQ31 Serine/threonine kinase | **F:** AAAAGGCACATAGCGTCCAT  **R:** AGTGGTGGAGACCAGGTTTG | 20  20 |
| 9. | P27357 Thaumatin-like protein PWIR2 precursor | **F:** GCAGCACCCAGGACTTCTAC  **R:** TGCGACGTATAGAGGCTTCA | 20  20 |
| 10. | P29114 LOX1 Lipoxygenase 1 | **F:** GATCGAGAGCAAGGTGGTG  **R:** TCAGATGGAGATGCTGTTGG | 19  20 |
| 11. | Q5NTH3 Shikimate kinase 2 | **F:** ATCCATACACAGCGGCTTTC  **R:** GTAGGGCCTCGACAGCAATA | 20  20 |
| 12. | P12940 Bowman-Birk trypsin inhibitor | **F:** GACCCATCCCTCAACGTCT  **R:** ACACCTGCTGGCGTATTCAT | 19  20 |
| 13. | Q6Z676 phi-1 ABA dehydration signaling | **F:** CACCTGTTCGACCTTGGTGT  **R:** GAAAGCCAGTGCAGCAATTT | 20  20 |
| 14. | P93671 XET Xyloglucan endotransglycosylase | **F:** GTGGGTGCAGAGCAACTACA  **R:** GGCGTAAATGCCAAAGAAGA | 20  20 |
| 15. | Q43210 PAL Phenylalanine ammonia lyase | **F:** ACCAGGGTAAGCACATCGAC  **R:** ATCTTTGGCAATGGCCTCTA | 20  20 |
| 16. | Q9P3N1 Allene oxide synthase (AOS) | **F:** CTTCACATCTAACGGGCATC  **R:** ATGGAGGTGCTTGAGACG | 20  18 |
| 17. | Q5ZD81 Calmodulin-like protein | **F:** AGGGAAGGGAAAGGATAAAGTG  **R:** CGACCTACAGACAGTACGC | 22  19 |
| 18. | Q6Z1A3 cinnamyl alcohol dehydrogenase 1 (CAD1) | **F:**GGAGGTTACATTACATTTGGAGAG  **R:** TGGAGTAGCATTGGACTATTGG | 24  22 |
| 19. | Q6YXE1 Cinnamoyl CoA reducatse 3 (CCR3) | **F:** TCATGGAGAAGAACAAGCAG  **R:** GAGGCGGGTGTAGAAGAG | 20  18 |
| 20. | Q7XN01 Isochorismate synthase (ICS) | **F:** GCCATTGCGGAGTCACAAG  **R:** TGGTTCGTCCTTCACTATGC | 19  20 |
| 21. | Q6I5G9 NADPH oxidase (NADPHOX) | **F:** TCATGGAGAAGAACAAGCAG  **R:** GAGGCGGGTGTAGAAGAG | 20  18 |
| 22. | TA1868 Wheat actin | **F:** GAGTCGGTGAAGGTTGTTTAC  **R:** CTTAGGCAGCGTTTGGAATAC | 21  21 |
